# Effect of moringa seed extract in chlorpyrifos-induced cerebral and ocular toxicity in mice

**DOI:** 10.3389/fvets.2024.1381428

**Published:** 2024-04-10

**Authors:** Ibtesam S. Alanazi, Ahmed E. Altyar, Mohamed Sayed Zaazouee, Alaa Ahmed Elshanbary, Abdel-Fattah M. Abdel-Fattah, Mohamed Kamel, Mai Albaik, Nehmat Ghaboura

**Affiliations:** ^1^Department of Biology, Faculty of Sciences, University of Hafr Al Batin, Hafr Al Batin, Saudi Arabia; ^2^Department of Pharmacy Practice, Faculty of Pharmacy, King Abdulaziz University, Jeddah, Saudi Arabia; ^3^Pharmacy Program, Batterjee Medical College, Jeddah, Saudi Arabia; ^4^Faculty of Medicine, Al-Azhar University, Assiut, Egypt; ^5^Faculty of Medicine, Alexandria University, Alexandria, Egypt; ^6^Pharmacology Department, Faculty of Veterinary Medicine, Suez Canal University, Ismailia, Egypt; ^7^Department of Medicine and Infectious Diseases, Faculty of Veterinary Medicine, Cairo University, Giza, Egypt; ^8^Department of Chemistry, Preparatory Year Program, Batterjee Medical College, Jeddah, Saudi Arabia; ^9^Pharmacy Practice Department, Pharmacy Program, Batterjee Medical College, Jeddah, Saudi Arabia

**Keywords:** moringa, chlorpyrifos, brain, eye, antioxidant, oxidative stress, organophosphate, insecticide

## Abstract

Chlorpyrifos (CPF) is one of the most commonly used organophosphosphate-based (OP) insecticides. Its wide use has led to higher morbidity and mortality, especially in developing countries. Moringa seed extracts (MSE) have shown neuroprotective activity, antioxidant, anti-inflammatory, and antibacterial features. The literature lacks data investigating the role of MSE against CPF-induced cerebral and ocular toxicity in mice. Therefore, we aim to investigate this concern. A total of 40 mature male Wistar Albino mice were randomly distributed to five groups. Initially, they underwent a one-week adaptation period, followed by a one-week treatment regimen. The groups included a control group that received saline, MSE 100 mg/kg, CPF 12 mg/kg, CPF-MSE 50 mg/kg, and CPF-MSE 100 mg/kg. After the treatment phase, analyses were conducted on serum, ocular, and cerebral tissues. MSE100 and CPF-MSE100 normalized the pro-inflammatory markers (interleukin-1β (IL-1β), interleukin-6 (IL-6), and tumor necrosis factor-alpha (TNF-α)) and AChE serum levels. CPF-MSE50 significantly enhanced these serum levels compared to CPF; however, it showed higher levels compared to the control. Moreover, the tissue analysis showed a significant decrease in oxidative stress (malondialdehyde (MDA) and nitric oxide (NO)) and an increase in antioxidant markers (glutathione (GSH), glutathione peroxidase (GSH-PX)), superoxide dismutase (SOD), and catalase (CAT) in the treated groups compared to CPF. Importantly, the significance of these effects was found to be dose-dependent, particularly evident in the CPF-MSE100 group. We conclude that MSE has a promising therapeutic effect in the cerebral and ocular tissues of CPF-intoxicated mice, providing a potential solution for OP public health issues.

## 1 Introduction

Organophosphosphate (OP)—based insecticides have been commonly employed in agricultural and household settings. Chlorpyrifos (CPF) [O, O-diethyl-o-(3, 5, 6-trichloro-2-pyridyl) phosphorothionate] is one of the most common OP-based insecticides, which is widely used due to its broad spectrum insecticidal activity (1–4). Its wide use has led to higher morbidity and mortality, especially in developing countries (5–7).

Chlorpyrifos affects many tissues, including neurological, ocular, cardiac, hepatic, and renal. It inhibits the acetylcholinesterase (AChE) enzyme in the neuronal synaptic clefts, which is essential for eliminating acetylcholine. AChE inhibition leads to acetylcholine (ACh) accumulation in those synaptic areas, causing toxic effects. Accumulated ACh causes neuronal, behavioral, and cognitive dysfunction, impaired memory, delayed neural development, and death (8–10). Moreover, CPF has been reported to interfere with the mitochondrial electron transport chain (ETC), increasing the production of reactive oxygen species (ROS). In addition, it interferes with the process important for eliminating ROS. CPF limits the antioxidant activities of certain enzymes, such as glutathione peroxidase (GPx), superoxide dismutase (SOD), and catalase (CAT). This in turn leads to oxidative stress, lipid peroxidation, and damage to the affected tissues (11, 12).

The use of plant extracts in medicine has been described for centuries (13). They provide a rich source of bioactive agents with notable effects (14–19). The Moringaceae family is a family of drought-resistant trees. *Moringa oleifera* (*M. oleifera*) is the most popular member of this family (15, 20, 21). This plant is characterized by containing various bioactive components in its seeds and leaves. It comprises several vitamins, minerals, and proteins such as vitamins A and C and iron, calcium, and potassium (22–25). *M. oleifera* extracts have shown neuroprotective activity, antioxidant, anti-inflammatory, and antibacterial features (25–28). Those features are attributed to containing polyphenols, terpenoids, and flavonoids, which regulate the antioxidant and detoxification activities (25, 26, 29).

Thereby *M. oleifera* seed extracts (MSE) have potential promising therapeutic effects against oxidative damage and OP toxicity (24). Although the known therapeutic effects of MSE, there is a shortage in the literature regarding its role in CPF intoxication. Hence, in this study, we aim to investigate the role of MSE on the CPF-intoxicated cerebral and ocular tissues of mice.

## 2 Methods

### 2.1 Chemical supplies

Pure moringa seed extract (MSE) and chlorpyrifos (CPF) were purchased from moringa sales at the National Research Centre (NRC), Egypt. The dried seeds were ground into fine powders and sieved. The enzyme-linked immunosorbent assay (ELISA) kits for pro-inflammatory cytokines, including interleukin-6 (IL-6), IL-1β, and tumor necrosis factor-alpha (TNF-α), were obtained from R&D (Mannheim, Germany) (Catalog # ARY005B). The AChE kit was obtained from Jiancheng Bioengineering Institute (Nanjing, China) (Catalog # A024). Kits for oxidative stress indicators and antioxidant status were purchased from Biodiagnostics Co. (Cairo, Egypt).

### 2.2 The study animals and design of the experiment

All procedures of our experiments were revised and approved by the Ethical Committee of the Faculty of Veterinary Medicine, Suez Canal University, Ismailia, Egypt (2023018).

A total of 40 mature male Wistar Albino mice (weighted 190 ± 10 g) were included in our experiments. They were obtained from the Egyptian Organization of Biological Products and Vaccines. Mice were cared for in laboratory chambers with suitable ventilation and temperature (25 ± 2°C) with a relatively humid atmosphere between 40 to 50% and under a 12-h cycle of light and dark. Additionally, they were supplied with enough food and water. For one week before the experiment, the mice were acclimated under these conditions.

After the one-week adaptation, mice were randomly distributed to one of five groups (eight mice in each group); the first group (control group) received saline as a control, group (2) received 100 mg/kg moringa seed extract (MSE), group (3) were administered 12 mg/kg chlorpyrifos (CPF) via oral gavage for 7 days, group (4) received 12 mg/kg CPF and 50 mg/kg MSE for 7 days and group (5) received 12 mg/kg CPF and 100 mg/kg MSE daily for 7 days.

### 2.3 Collection of blood samples and preparation of tissues

Blood samples were obtained from the retro-orbital venous plexus on the 15th day of the experiment and were centrifuged at 3000 g for 15 min. Then the obtained serum was kept at −20°C for further testing. All mice were sacrificed using isoflurane after blood sample collection. The cerebral and ocular tissue samples were obtained from all animals, cleaned of blood clots with saline and purified water then traditionally manually dissected. Then, all dissected samples were homogenized in 5–10 mL of ice-cold buffer per gram of tissue. After that, the tissues were centrifuged at 5000 rpm for 30 min. The formed supernatant was collected in tubes and stored at −80°C for spectrophotometry.

### 2.4 Assessment of AChE levels

The AChE levels were measured using colorimetric kits that use a specific enzymatic reaction to measure AChE levels in serum samples. Detailed assay procedures were strictly followed according to the manufacturer’s instructions. Specifically, the assays were guided by the Lowry assay (30) for protein concentration measurement.

### 2.5 Assessment of the pro-inflammatory mediators

We used the ELISA kits to assess the pro-inflammatory cytokines, including IL-6, IL-1β, and TNF-α, following the protocol provided by R&D (Mannheim, Germany).

### 2.6 Statues of tissue antioxidant and oxidative stress markers

Nitric oxide (NO) and malondialdehyde (MDA) were the markers for lipid peroxidation and were assessed using spectrophotometry techniques. NO was assessed as described by Green et al. (31) while MDA was estimated as described by Mihara et al. (32). Tissue anti-oxidant markers included glutathione (GSH), GSH peroxidase (GSH-Px), catalase (CAT), and superoxide dismutase (SOD) and were assessed using methods described by Beutler et al. (33), Paglia et al. (34), Aebi (35), and Nishikimi et al. (36) respectively.

### 2.7 Statistical analysis

Statistical analysis was done using the Statistical Package for social sciences (SPSS) 26.0. The Shapiro–Wilk test was employed to assess the normal distribution of the data. The statistical significance of the results was determined using the one-way analysis of variance (ANOVA) test. Tukey’s multiple range test was used for individual comparisons. Data were presented as mean and standard error (SE). Data were considered significant if the *p*-value was <0.05.

## 3 Results

### 3.1 Role of moringa seed extract on serum pro-inflammatory cytokines and acetylcholinesterase in CPF-intoxicated mice

The control group showed significantly lower levels than the CPF group regarding the IL-1β, IL-6, and TNF-α (33.7, 28.2, and 28.3%, respectively). Similarly, the MSE100 group showed significantly lower levels of IL-1β, IL-6, and TNF-α than the CPF group (31, 27.1, and 27.2%, respectively). Moreover, compared with the CPF group, the CPF-MSE50, and CPF-MSE100 showed significantly lower levels of IL-1β (63.2 and 37.5%, respectively), IL-6 (60 and 33.2%, respectively) and TNF-α (52.4 and 30.5%, respectively). However, the levels of the inflammatory markers in the MSE100 and CPF-MSE100 did not significantly differ from the control group. Additionally, the CPF-MSE50 showed significantly higher cytokine levels than the control group.

**Figure 1:**
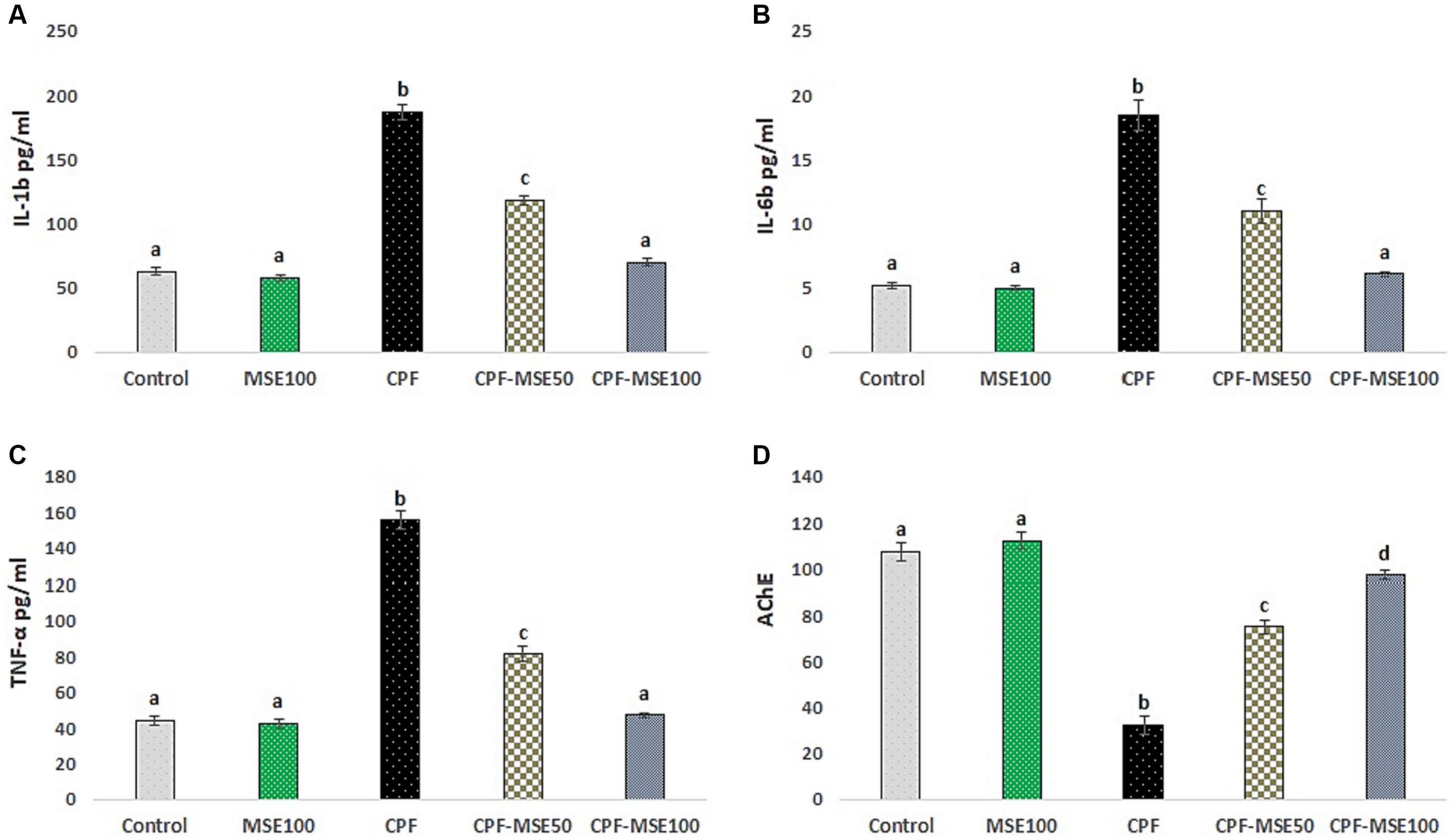
Effect of MSE on serum levels in CPF-intoxicated mice. This figure depicts the impact of Moringa seed extract (MSE) on serum levels in mice intoxicated with Chlorpyrifos (CPF). **(A)** refers to interleukin-1β (IL-1β); **(B)** refers to interleukin-6 (IL-6); **(C)** refers to tumor necrosis factor-alpha (TNF-α); **(D)** refers to acetylcholinesterase (AChE). Serum levels of pro-inflammatory markers (IL-1β, IL-6, TNF-α, and AChE) were assessed in five experimental groups: Control negative, MSE 100 mg/kg, CPF 12 mg/kg, CPF-MSE 50 mg/kg, and CPF-MSE 100 mg/kg. Values having different alphabetic superscripts are significantly different (*p* < 0.05).

Regarding acetylcholinesterase (AChE), the control group showed significantly higher levels than the CPF group (3.3%). The CPF group also showed significantly lower levels than the other groups (control; 3.3%, MSE100; 3.4%, CPF-MSE50; 2.3%, and CPF-MSE100; 3%). However, there was no significant difference between MSE100, CPF-MSE100, and control groups (Table 1; Figure 1).

**Table 1:** Role of MSE on serum pro-inflammatory cytokines and acetylcholinesterase in CPF-intoxicated mice.

Parameters	Groups				
	Control	MSE100	CPF	CPF-MSE50	CPF-MSE100
IL-1β (pg/ml)	63.04^a^ ± 3.03	58.02^a^ ± 2.47	187.2^b^ ± 5.74	118.23^c^ ± 3.42	70.11^a^ ± 3.21
IL-6 (pg/ml)	5.2^a^ ± 0.22	5.0^a^ ± 0.19	18.45^b^ ± 1.14	11.03^c^ ± 0.93	6.12^a^ ± 0.17
TNF-α (pg/ml)	44.1^a^ ± 2.65	42.43^a^ ± 2.19	155.75^b^ ± 5.11	81.57^c^ ± 4.41	47.44^a^ ± 1.29
AChE (U/mL)	107.29^ad^ ± 3.82	112.05^a^ ± 3.62	32.55^b^ ± 4.07	75.28^c^ ± 2.98	97.28^d^ ± 2.07

MSE100; Moringa seed extract 100 mg, MSE50; Moringa seed extract 50 mg, CPF; chlorpyrifos, IL-1β; interleukin-1β, IL-6; interleukin-6, TNF-α; tumor necrosis factor-alpha, AChE; acetylcholinesterase. Data are expressed as mean ± standard error (SE). Values with different alphabetic superscripts within the same row differ significantly (*p* < 0.05).

### 3.2 Effect of MSE on ocular tissue oxidative stress and antioxidant levels in CPF-intoxicated mice

**Figure 2:**
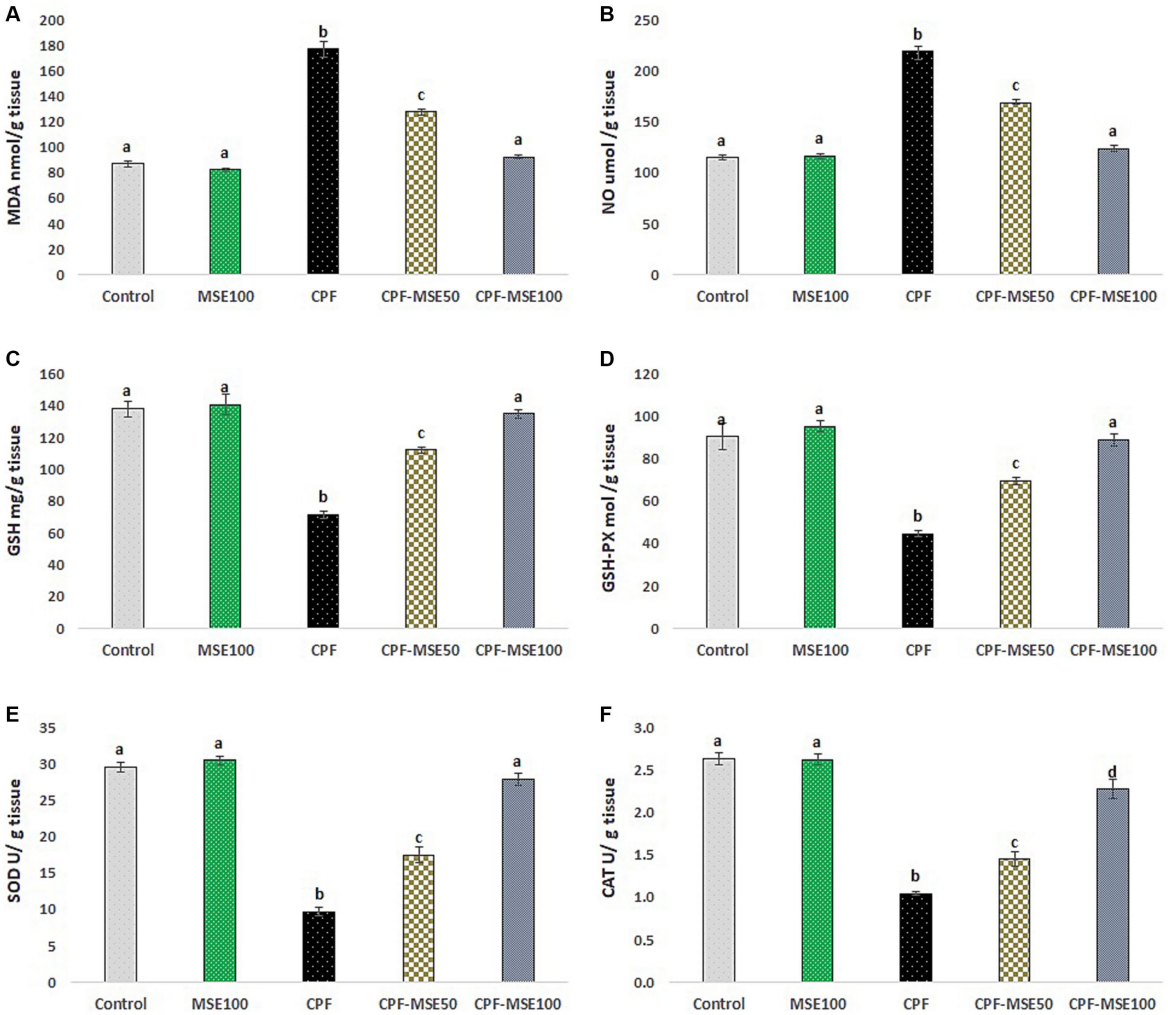
Effect of MSE on ocular tissue oxidative stress and antioxidants levels in CPF-intoxicated mice. **(A)** refers to malondialdehyde (MDA); **(B)** refers to nitric oxide (NO); **(C)** refers to glutathione; **(D)** refers to glutathione peroxidase (GSH-PX); **(E)** refers to superoxide dismutase (SOD), and **(F)** refers to catalase (CAT). This figure presents the impact of Moringa Seed Extract (MSE) on oxidative stress (MDA and NO) and antioxidant levels (GSH-PX, SOD and CAT) in the ocular tissues of mice exposed to Chlorpyrifos (CPF). Values having different alphabetic superscripts are significantly different (*p* < 0.05).

Compared with the CPF group, the MSE100, CPF-MSE50, and CPF-MSE100 showed significantly lower levels of MDA (46.5, 72.1, and 52.1%, respectively) and NO (53.3, 77.1, and 56.5%). On the other hand, the MSE100, CPF-MSE50, and CPF-MSE100 showed significantly higher levels of GSH (50.8, 64, 53%), GSH-PX (47, 64.3, and 50.2%, respectively), SOD (31.7, 55.4, and 34.7%, respectively), and CAT (40.1%, 72.4, and 46.3%, respectively) when compared with CPF group (Table 2; Figure 2). However, MSE100, CPF-MSE100, and the control group significantly differ regarding all oxidative stress and antioxidant parameters except for CAT. The CAT level was significantly higher in the control group compared with the CPF-MSE100 group.

**Table 2:** Effect of MSE on ocular tissue oxidative stress and antioxidant levels in CPF-intoxicated mice.

Parameters	Groups				
	Control	MSE100	CPF	CPF-MSE50	CPF-MSE100
MDA (nmol/g) tissue	86.86^a^ ± 2.77	82.41^a^ ± 1.525	177.04^b^ ± 6.11	127.68^c^ ± 2.41	92.32^a^ ± 1.6
NO (μmol/g) tissue	114.58^a^ ± 1.73	116.58^a^ ± 2.22	218.75^b^ ± 8.28	168.69^c^ ± 1.69	123.65^a^ ± 2.7
GSH (mg/g) tissue	138.04^a^ ± 4.92	140.42^a^ ± 6.45	71.33^b^ ± 2.61	111.97^c^ ± 2.18	134.97^a^ ± 2.73
GSH-PX (mol/g) tissue	90.42^a^ ± 6.08	95.09^a^ ± 2.6	44.62^b^ ± 1.27	69.44^c^ ± 1.6	88.94^a^ ± 2.94
SOD (U/g) tissue	29.56^a^ ± 0.6	30.49^a^ ± 0.6	9.67^b^ ± 0.55	17.47^c^ ± 1.08	27.9^a^ ± 0.84
CAT (U/g) tissue	2.63^a^ ± 0.07	2.62^a^ ± 0.06	1.05^b^ ± 0.03	1.45^c^ ± 0.09	2.27^d^ ± 0.11

MSE; Moringa seed extract, CPF; chlorpyrifos, MDA; malondialdehyde, NO; nitric oxide, GSH; glutathione, GSH-PX; glutathione peroxidase, SOD; superoxide dismutase, CAT; catalase. Data are expressed as mean ± standard error (SE). Values having different alphabetic superscripts within the same row are significantly different (*p* < 0.05).

### 3.3 Effect of MSE on cerebral tissue oxidative stress and antioxidant levels in CPF-intoxicated mice

**Figure 3:**
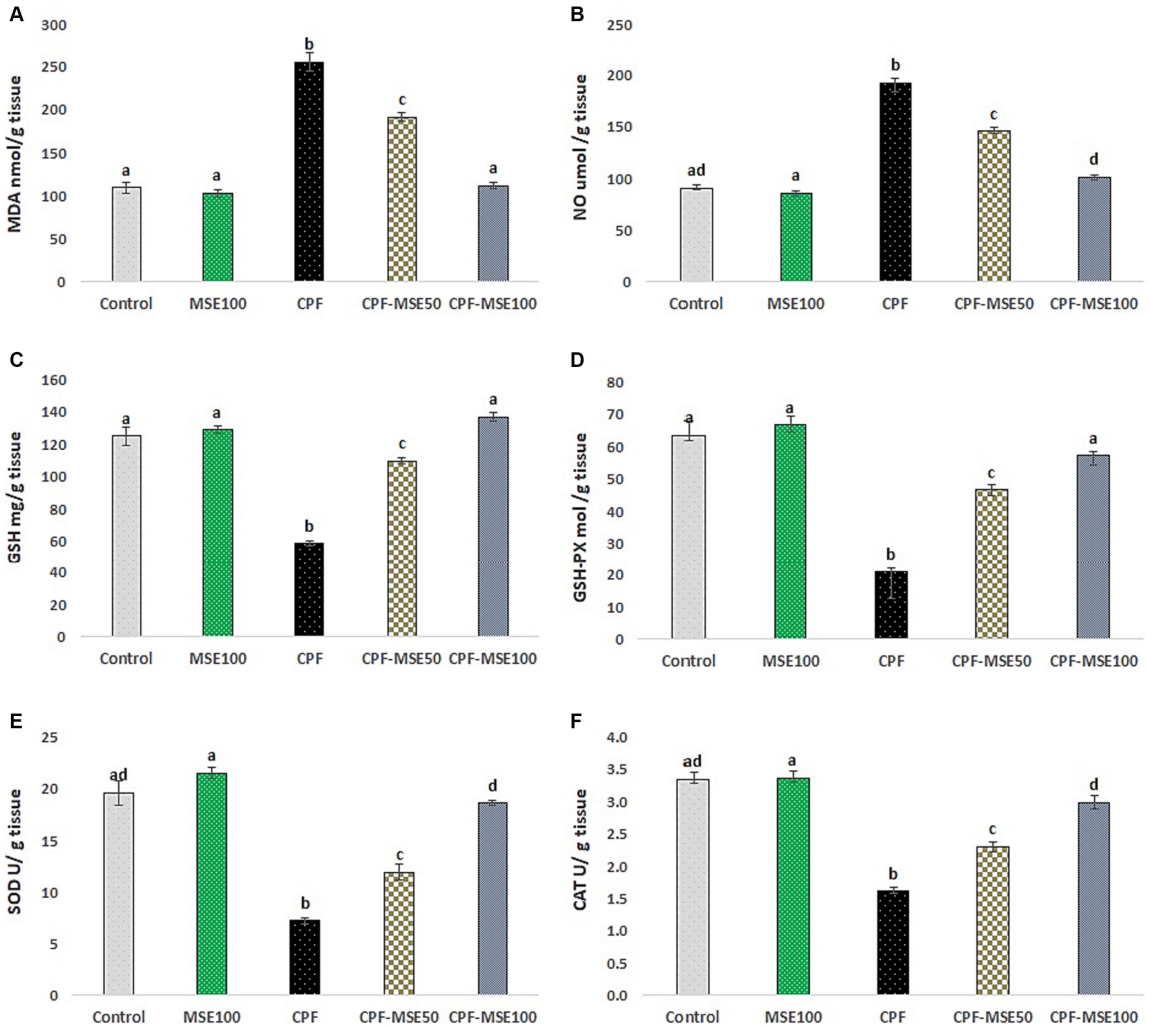
Effect of MSE on cerebral tissue oxidative stress and antioxidant levels in CPF-intoxicated mice. **(A)** refers to malondialdehyde (MDA); **(B)** refers to nitric oxide (NO); **(C)** refers to glutathione; **(D)** refers to glutathione peroxidase (GSH-PX); **(E)** refers to superoxide dismutase (SOD), and **(F)** refers to catalase (CAT). This figure presents the impact of Moringa Seed Extract (MSE) on oxidative stress (MDA and NO) and antioxidant levels (GSH, GSH-PX, SOD and CAT) in the cerebral tissue of mice exposed to Chlorpyrifos (CPF). Values having different alphabetic superscripts are significantly different (*p* < 0.05).

**Figure 4:**
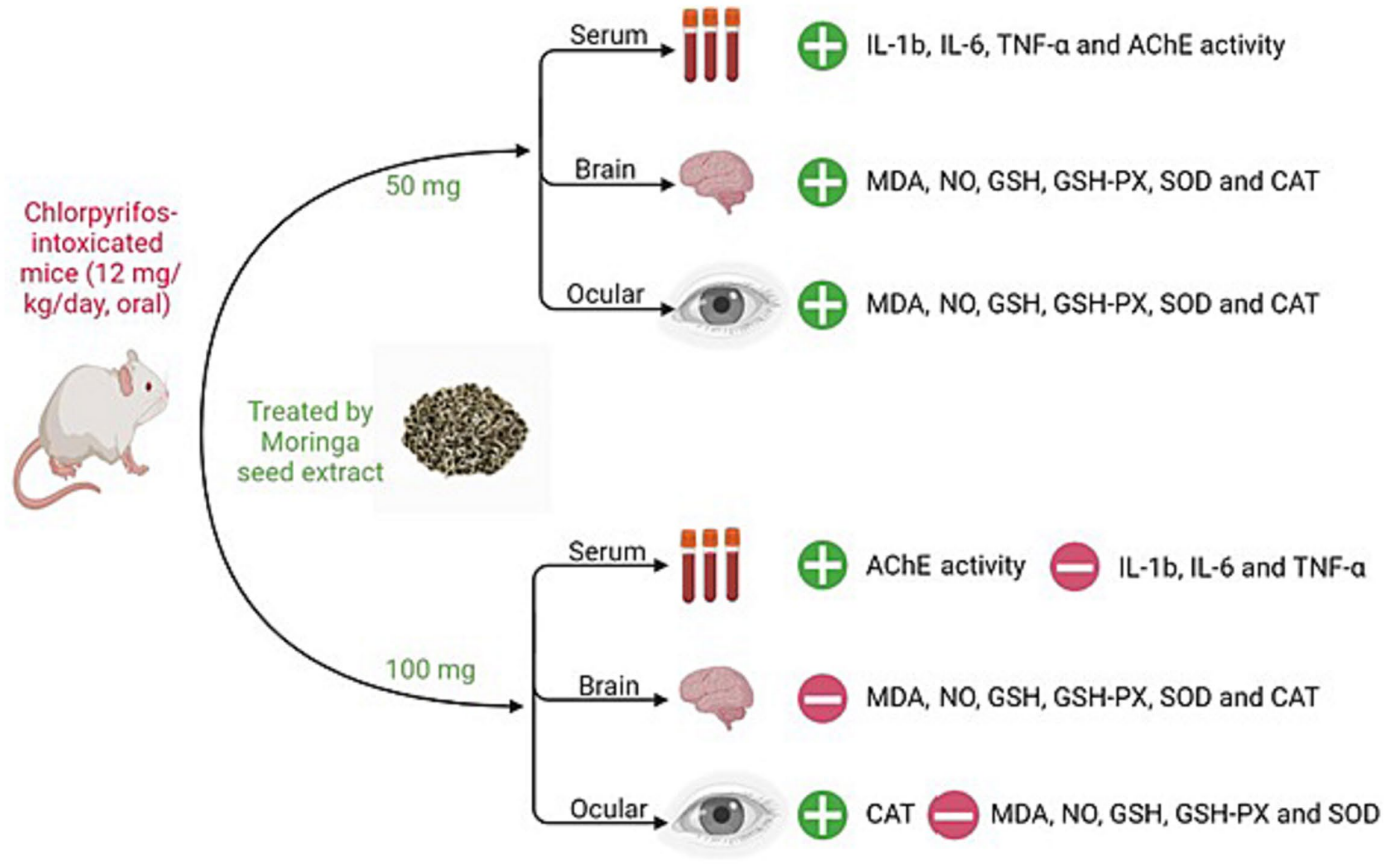
Summary of MSE effect on cerebral and ocular toxicity induced by CPF in mice. IL-1β; interleukin-1β, IL-6; interleukin-6, TNF-α; tumor necrosis factor-alpha, AChE; acetylcholinesterase, MDA; malondialdehyde, NO; nitric oxide, GSH; glutathione, GSH-PX; glutathione peroxidase, SOD; superoxide dismutase, CAT; catalase.

Compared with the CPF group, the MSE100, CPF-MSE50, and CPF-MSE100 groups showed significantly lower levels of MDA (40.3, 75, and 44%, respectively), and NO (44.6, 76, and 53% respectively). On the other hand, the MSE100, CPF-MSE50, and CPF-MSE100 showed significantly higher levels of GSH (45.3, 53.3, and 43%, respectively), GSH-PX (31.6, 45.2, and 37% respectively), SOD (33.7, 61, and 39% respectively), and CAT (48.1, 70.1, and 54.2% respectively) compared with CPF group (Table 3; Figure 3). On the other hand, MSE100 and CPF-MSE100 did not significantly differ regarding all oxidative stress markers and antioxidants. In addition, MSE100 and CPF-MSE100 showed significantly better results compared with CPF-MSE 50 (Figure 4).

**Table 3:** Effect of MSE on cerebral tissue oxidative stress and antioxidant levels in CPF-intoxicated mice.

Parameters	Groups				
	Control	MSE100	CPF	CPF-MSE50	CPF-MSE100
MDA (nmol/g) tissue	109.45^a^ ± 6.62	103.07^a^ ± 4.6	255.76^b^ ± 10.19	191.8^c^ ± 4.86	112.07^a^ ± 3.35
NO (μmol/g) tissue	91.03^ad^ ± 3.44	85.89^a^ ± 1.81	192.4^b^ ± 4.8	146.07^c^ ± 3.5	101.53^d^ ± 1.75
GSH (mg/g) tissue	125.15^a^ ± 5.51	129.19^a^ ± 2.37	58.53^b^ ± 1.49	109.72^c^ ± 1.94	136.93^a^ ± 2.58
GSH-PX (mol/g) tissue	63.59^a^ ± 4.44	66.85^a^ ± 2.64	21.1^b^ ± 1.18	46.65^c^ ± 1.82	57.26^a^ ± 1.3
SOD (U/g) tissue	19.62^ad^ ± 1.2	21.54^a^ ± 0.51	7.25^b^ ± 0.29	11.93^c^ ± 0.78	18.62^d^ ± 0.26
CAT (U/g) tissue	3.35^ad^ ± 0.11	3.37^a^ ± 0.11	1.62^b^ ± 0.06	2.31^c^ ± 0.08	2.99^d^ ± 0.1

MSE; Moringa seed extract, CPF; chlorpyrifos, MDA; malondialdehyde, NO; nitric oxide, GSH; glutathione, GSH-PX; glutathione peroxidase, SOD; superoxide dismutase, CAT; catalase. Data are expressed as mean ± standard error (SE). Values with different alphabetic superscripts within the same row differ significantly (*p* < 0.05).

## 4 Discussion

In this study, we aimed to investigate the protective role of MSE against CPF toxicity. We found that MSE has a promising therapeutic effect in the cerebral and ocular tissues of CPF-intoxicated mice. Compared with the CPF group, the MSE 100, CPF-MSE50, and CPF-MSE100 groups significantly elevated the AChE levels. However, the MSE100 and CPF-MSE100 AChE levels did not significantly differ from the values of the control group. But they were still significantly better than the CPF group. Moreover, MSE, MSE 100, CPF-MSE50, and CPF-MSE100 groups showed a significant reduction in the serum levels of the inflammatory mediators compared to the CPF group. In this study, MSE decreased the oxidative stress markers (MDA and NO) levels and elevated the levels of antioxidant agents (GSH, GSH-PX, SOD, and CAT) in the ocular and cerebral tissues of mice. MSE50, MSE100, and CPF-MSE 100 groups significantly enhanced oxidative stress compared with the CPF group. However, MSE100 and CPF-MSE100 showed significantly better results compared with CPF-MSE 50.

Notably, the values of oxidative stress markers were higher in the cerebral tissue compared with the ocular tissue. In addition, cerebral tissue showed lower levels of antioxidants, indicating their consumption by the cerebral tissue more than the ocular tissue. This points to the significant oxidative stress the brain tissue endures. Cerebral tissue is characterized by higher liability to oxidative damage induced by ROS (8). This is attributed to higher energy demand and lower anti-oxidant levels such as SOD and CAT, in addition to, higher content of polyunsaturated fatty acids (PUFA), proteins, and nucleic acids (24, 37).

Given the findings indicating decreased serum levels of inflammation and markers of oxidative stress, it is important to mention the fundamental mechanisms through which MSE exerts its protective effects. MSE’s compounds demonstrate anti-inflammatory effects by inhibiting pro-inflammatory enzymes such as cyclooxygenase and lipoxygenase, as observed with quercetin and kaempferol. Additionally, MSE regulates cytokine production by modulating signaling pathways like nuclear factor-kappa B (NF-kappa B), thereby decreasing the production of pro-inflammatory cytokines such as TNF-α and IL-1β. Furthermore, MSE contains flavonoids and polyphenols known for their antioxidant properties, which contribute to reducing oxidative stress and inflammation (38, 39).

Similarly, it has been reported that chronic OP insecticide exposures lead to chronic ocular damage through OP-induced retinal apoptosis (40–44). Retina easily suffers from CPF-induced damage. Normally, it is subjected to severe oxidative stress resulting from the high metabolic activities stimulated by light exposure. Moreover, retinal oxidative stress has been related to a variety of retinal pathological conditions. However, the effect of CPF on eye structures other than the retina needs further investigation (44, 45).

CPF as one of the OP compounds interferes with macromolecule production such as DNA, RNA, and proteins. In addition, it impairs normal neuronal development and interferes with the normal process of neurotransmission cascade through the affection of several enzymes important for signal transduction (8, 46, 47). In addition, CPF inhibits the AChE enzyme and has been linked to oxidative damage of human and animal tissues (9–12, 48).

Oxidative tissue damage is attributed to the accumulation of reactive oxygen species (ROS); the metabolic products of the energy production cascade. Under normal circumstances, it can be controlled by the scavenger action of antioxidant enzymes like GSH, GSH-PX, SOD, and CAT. The imbalance between ROS and anti-oxidants results in the accumulation of harmful ROS leading to significant tissue damage and cellular apoptosis (49, 50).

Our findings point to the role of oxidative damage and reactive oxygen species (ROS) in cerebral and ocular tissues of CPF-intoxicated mice. This is evidenced by the significantly higher levels of oxidative stress markers and the decreased level of the antioxidant agents in the CPF-intoxicated groups. As a result of ROS activity, the antioxidant enzymes were significantly consumed with significantly higher values of MDA and NO in the CPF group compared to the control, MSE, and CPF-MSE 100 groups.

Our results also indicate a probable dose-dependent pattern of MSE. This is evidenced by the significant reduction of the inflammatory mediators, oxidative stress marker, and the significant elevation of AChE, and the antioxidant agents in the MSE100 and CPF-MSE100 compared with the CPF-MSE50 group. Additionally, MSE100 and CPF-MSE100 showed similarity to the baseline values of the control group with no statistically significant difference. This points to the significant role of MSE that achieves tissue homeostasis even in CPF-intoxicated cerebral and ocular tissues of mice. Furthermore, our results confirm what has been previously reported in the literature regarding its anti-oxidant (51–55), anti-inflammatory (24, 56, 57), and AChE-enhancing features (24, 28, 58). However, the latter is debatable.

The therapeutic properties of MSE are ascribed to the potent constituents of its seeds and leaves, which include flavonoids, phenolics, carotenoids, and vitamins A and C, alongside essential minerals such as iron, calcium, and potassium (15, 23, 25, 59, 60). Additionally, MSE is rich in bioactive phytochemicals like niazirin, niazimicin, β-sitosterol, and 4(alpha-L-rhamnosyloxy) benzyl isothiocyanate, each contributing to its therapeutic potential (61). For instance, niazirin is recognized for its strong antioxidant activity (62); niazimicin has neuroprotective features (63); β-sitosterol exhibits anti-adipogenic activities (64); and the α rhamnosyloxy has been reported to attenuate spinal cord injury-associated damage (65). However, the exact mechanism of its protective role has not been established yet. In previous studies, the anti-oxidant power of MSE was assessed by defining the total amounts of phenols, flavonoids, and tannin contents in addition to defining the scavenging and reducing powers of the diphenyl picrylhydrazyl (DPPH), azinobis-ethylbenzothiazoline-sulfonic acid (ABTS), and nitric oxide (NO) (53). The DPPH, ABTS, and NO scavenging activities were attributed to the phenolic and flavonoid contents of the moringa seed water extracts (53, 66).

Considering our results alongside prior research, MSE exhibits several protective and therapeutic effects against OP toxicity through its antioxidant, anti-inflammatory, and AChE inhibitory actions. These findings indicate a possible avenue for addressing OP-related public health concerns. Consequently, we recommend further exploration into the protective potential of MSE in human populations.

## 5 Conclusion

Our study findings highlight the promising therapeutic effects of MSE in ameliorating CPF toxicity in mice. MSE administration resulted in significant enhancements in AChE activity and antioxidant functions while concurrently reducing levels of inflammatory mediators and oxidative stress markers in both cerebral and ocular tissues of CPF-intoxicated mice.

In conclusion, our study highlights MSE as a promising natural intervention for mitigating CPF toxicity, offering insights into its therapeutic mechanisms and suggesting avenues for future research. By embracing these insights and addressing the identified research gaps, we can advance our understanding of MSE’s potential in alleviating pesticide-induced toxicity and contribute to the development of effective therapeutic interventions based on natural compounds like MSE.

As a limitation of the current study, specific molecular pathways should be deeply involved in its preventive and therapeutic effects, and assess its safety and efficacy in clinical settings.

## Data availability statement

The original contributions presented in the study are included in the article/supplementary material, further inquiries can be directed to the corresponding author.

## Ethics statement

The animal study was approved by all procedures of our experiments were revised and approved by the Ethical Committee of the Faculty of Veterinary Medicine, Suez Canal University, Ismailia, Egypt (2023018). The study was conducted in accordance with the local legislation and institutional requirements.

## Author contributions

IA: Formal analysis, Writing – review & editing, Conceptualization, Investigation, Methodology, Project administration, Software, Validation, Writing – original draft, Funding acquisition, Supervision, Visualization. AA: Conceptualization, Formal analysis, Funding acquisition, Investigation, Methodology, Project administration, Supervision, Validation, Visualization, Writing – review & editing. MZ: Formal analysis, Investigation, Methodology, Visualization, Data curation, Software, Writing – original draft. AE: Data curation, Formal analysis, Investigation, Methodology, Software, Visualization, Writing – original draft, Project administration. A-FA-F: Data curation, Methodology, Software, Visualization, Writing – original draft, Validation. MK: Data curation, Methodology, Software, Visualization, Writing – original draft. MA: Software, Writing – original draft, Conceptualization, Investigation, Project administration, Validation. NG: Conceptualization, Formal analysis, Investigation, Methodology, Project administration, Supervision, Validation, Visualization, Writing – review & editing.
